# Clinical and Biological Validation of an Allogeneous Cancellous Bone Block for Alveolar Maxillary Ridge Reconstruction: A Case Series

**DOI:** 10.3390/dj12020042

**Published:** 2024-02-19

**Authors:** Alexandre Perez, Elena Pierantozzi, Roberto Di Felice, Tommaso Lombardi

**Affiliations:** 1 Unit of Oral Surgery and Implantology, Division of Oral and Maxillofacial Surgery, Department of Surgery, Geneva University Hospitals, Faculty of Medicine, University of Geneva, 1205 Geneva, Switzerland; 2 Private Practice, Studio Roberto di Felice, Viale Buozzi 6, 63074 San Benedetto del Tronto, Italy; 3 Unit of Oral Medicine and Oral Maxillofacial Pathology, Division of Oral and Maxillofacial Surgery, Department of Surgery, Geneva University Hospitals, Faculty of Medicine, University of Geneva, 1205 Geneva, Switzerland; tommaso.lombardi@unige.ch

**Keywords:** block augmentation, lateral augmentation, allograft, human histology, Maxgraft, bone block

## Abstract

This exploratory case series clinically and histologically investigated the performance of allogeneic cancellous freeze-dried bone allograft (FDBA) bone blocks (Maxgraft^®^) for the lateral augmentation of local alveolar defects in the posterior maxilla as part of two-staged implant therapy. Five patients receiving eight implants 5 months after block augmentation with a follow-up period of up to 3 years were documented and analyzed. Horizontal alveolar dimensions before and 5 months after block augmentation were quantified using CBCT. Radiographic marginal bone level changes were quantified at implant placement, loading, and 1 year post-placement. Graft integration and resorption were histologically qualitatively evaluated from core biopsies retrieved at implant placement. Block augmentations resulted in a pronounced horizontal median bone gain of 7.0 (5.5 to 7.8) mm. Marginal implant bone levels in block-augmented bone remained constant over the 1 year follow-up period. Block grafts appeared histologically well integrated. Histologic analysis also revealed signs of progressive resorption and new bone formation at the lateral aspects of the grafts. The results of this case series support using Maxgraft^®^ cancellous FDBA blocks as suitable materials for the lateral augmentation of local alveolar defects.

## 1. Introduction

Tooth loss, implant failure, trauma, and inflammatory processes are often associated with the loss of alveolar bone and may render implant treatments increasingly difficult [1]. Implant treatments in affected patients may require preceding or simultaneous bone-augmentative procedures [2]. Among the different treatment options, block grafts have recently attracted considerable interest due to their ability to predictably increase the horizontal alveolar dimension [3,4,5,6]. Recent systematic reviews and individual studies have reported the superior capacity to maintain volume and reduced risk of graft dislocation of block grafting compared to guided bone regeneration (GBR) using particulate material [2,7,8].

Autogenous grafts are generally considered the gold-standard bone-augmentative material due to their combination of osteogenic, osteoconductive, and osteoinductive proprieties. Their use is, however, associated with additional comorbidities, treatment risks, and the surgical trauma of a second surgical harvesting site. These disadvantages have recently motivated the use of allogenic block graft materials as viable and readily available alternatives [9].

Freeze-dried bone allografts (FDBAs) and demineralized FDBAs (DFDBAs) represent the most common types of allograft materials. Both types have been described for horizontal ridge augmentation procedures [10,11]. A recent consensus statement summarized that allogeneic bone grafts bear the collagenous matrix and proteins of natural bone and provide similar mechanical and handling properties compared to autologous bone while omitting viable osteogenic cells [12]. The current evidence and conclusions from recent systematic reviews suggest that the reconstruction of the atrophied alveolar crest using allogeneic bone block grafts may result in overall high success, minimal resorption, similar implant survival, and fewer postoperative complication rates compared to autogenous block grafts [13,14,15]. Block allografts also combine the advantage of equal or superior clinical performance compared to particulate graft materials while preventing the patient from experiencing potential comorbidities and risks associated with autologous graft harvesting [2,7,8].

The use of allografts is generally considered safe. Prospective clinical studies have failed to show disease transmission via allografts [16]. However, the potential risk of such transmissions, unwanted immune reactions, and type-specific material property differences remain controversial [12,17]. These potential drawbacks have been attributed to significant variations in production methods and a general lack of standardized purification processes for individual allograft types. Strong variabilities in constituents and potentially immuno- or pathogenic tissue remnants in bone blocks have been reported [17,18]. Their potential to elicit immune reactions or influence the regenerative capacity remains unclear [17,19]. The variabilities with regard to composition and purification processes likewise suggest a necessity for the individual clinical and biological validation of single-allograft materials [12,17,18,20].

Maxgraft^®^ block grafts (Botiss Biomaterials GmbH, Berlin, Germany) are allogeneic cancellous FDBAs derived from the femoral heads of living human donors from German, Austrian, and Swiss hospitals [20]. The material undergoes physical and chemical purification steps, including solvent extraction and oxidative chemical treatment, followed by freeze-drying and sterilization to remove cells and antigens, inactivate potential viruses and bacteria, and render the material sterile for clinical use [21,22]. Solakoglu et al. recently reported significant differences between two similar types of granular allografts, comprising Maxgraft granules, in augmenting and maintaining alveolar dimensions as part of a two-staged single implant restorative procedure while failing to show differences as part of an earlier human immunohistochemical and histometric study [6,23]. This controversy emphasizes the limited comparability between individual allografts and suggests a requirement for the material- and type-specific characterization and clinical performance validation of individual allografts.

Fretwurst et al. and Lorenz et al. recently reported on the in vitro histological and biochemical analysis of bone blocks of the study graft material used herein. Both authors reported on the presence of cell remnants comprising mono- and multinucleate cells and osteocytes in the grafts and on the presence of fibrous and fatty tissue [17,20]. Fretwurst et al. extracted significant amounts of DNA from the material [17]. Although the effect of these tissue remnants on the clinical performance and safety of these materials remains unclear, results from implantation studies with xenografts suggest that tissue remnants may elicit non-physiological pro-inflammatory tissue responses [24,25,26].

This case series described the clinical performance of Maxgraft allogenous cancellous block grafts for the lateral augmentation of posterior maxillary alveolar ridge defects and combined lateral and sinus-augmentative procedures. The study combined clinical, radiographic, and histological methods aiming to provide a qualitative and quantitatively comprehensive picture of the material’s clinical and biological performance in an exploratory case series setting, properties that have hitherto not been documented for the study material.

## 2. Materials and Methods

### 2.1. Case Series Setup

This case series documented the treatment of five patients with a total of 8 implants following bone augmentation as part of routine dental implant therapy at the University Hospital Geneva (Switzerland) between 2019 and 2021. Written informed consent was obtained from all patients to treat, document, and publish the treatment-related data. Treatments and reporting adhered to the Helsinki Declaration of Ethical Principles by the World Medical Association. The presented exploratory treatment scheme was limited to five patients. It did not require additional surgical sessions compared to routine treatment, classifying it as exempt from submission to the Institution’s Ethical Review Board (IRB) (Commission Cantonale d’Ethique de la Recherche sur l’être humain, Geneva, Switzerland (CCER)). 

### 2.2. Patient Selection

Treated patients were between 27 and 68 years old (3 men and 2 women) and demonstrated physical and psychological ability to undergo implant therapy (American Academy of Anesthesiologists class I or II). All patients were Cone Beam Computer Tomography (CBCT) (Newtom Go, Newtom Cefla, Bologna, Italy)-diagnosed with moderate to severe local horizontal bone deficiencies. Bone deficiencies prevented implant placement at adequate prosthetic positions as the main criterium for case selection and treatment described in the present report. None of the patients displayed any systemic medical condition that was contributory to bone augmentation and implant therapy. All patients were non-smokers and in good health. All patients were treated according to a similar 2-stage surgical procedure comprising augmentation with allogeneic bone blocks (Maxgraft^®^, Botiss Biomaterials, Zossen, Germany) and implant placement after a 5-month healing period.

### 2.3. Bone Block Augmentation Procedure

Bone augmentations and implant placement were performed under local anesthesia (4% articaine with 1:100’000 adrenaline–Ubistesin™ Forte–3M ESPE, Stuttgart, Germany) and after complete exposure of the osseous defect by raising a full-thickness flap following midcrestal incision, intrasulcular incisions in adjacent dentate areas, and vertical releasing incisions. Co-amoxicillin (Co-Amoxi-Mepha, Mepha Pharma, Aesch, Switzerland) was administered one hour prior to surgery (2 g) and continued for 6 days after surgery (2 × 1 g daily). 

**Figure 1:**
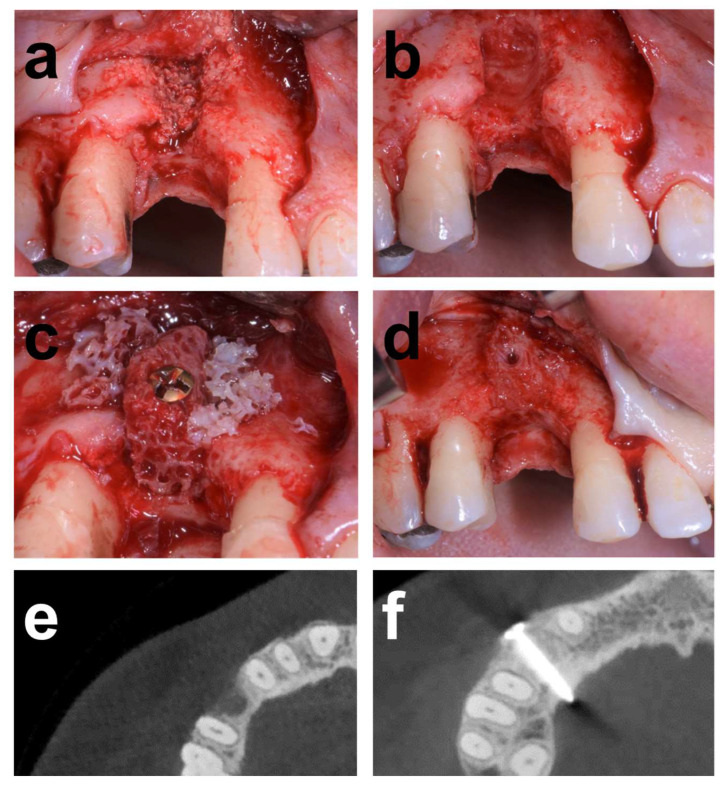
Treatment illustration of Case 1 patient presenting a combined alveolar defect resulting from removing a failing implant due to unsuccessful secondary peri-implant therapy involving bone augmentation. (**a**) Baseline situation following implant removal and healing, after mucoperiosteal flap elevation, and (**b**) after thorough debridement to remove residual bone graft material from the previous augmentation. The resulting combined defect displayed horizontal and vertical dimensions of 5 and 11 mm, respectively. (**c**) Situation after lateral bone block augmentation and fixation. Bone block contours were adapted to the alveolar contours, and bone chips from the trimming procedure were used to augment the lateral aspects of the block graft. (**d**) Vestibular aspect of the defect after 5 months of healing and exposure in preparation for implant placement. (**e**,**f**) Transversal 2D CBCT sections of the horizontal alveolar defect after implant removal and 5 months after block augmentation, respectively.

Defects were thoroughly debrided from granulation tissue (Figure 1). Subsequently, MaxGraft^®^ blocks (20 × 10 × 10 mm, 1xblock) were adapted according to the defect sizes and local contours of surrounding vital bone and secured into the defects using a 12 mm fixation screw (Cortex Screw 1.5 mm, 10 mm, Synthes^®^, Solothurn, Switzerland). Lateral aspects of the defect were augmented using allogenic bone chips resulting from trimming the blocks. 

Two patients received additional sinus-augmentative procedures. Maxillary sinus floor augmentations were performed following the lateral window technique under local anesthesia. In brief, lateral sinus windows were prepared using a round bur and intra-sinusoidal compartments for bone block placement were prepared through careful dorsocranial displacement of the Schneiderian membrane. Allogenious bone blocks were adapted in shape and size, positioned against the sinus floor, and secured by placing an intercortical fixation screw.

All augmentations were covered with a resorbable collagen membrane (Osteobiol^®^ Evolution, Technoss, Turin, Italy), followed by primary tension-free wound closure using Supramid 4.0 sutures (B.Braun^®^, Melsungen, Germany). Nonsteroidal analgesics (Irfen 600 mg and Dafalgan 1000 mg) were prescribed. Patients were advised to follow routine precautionary measures, including a soft cold diet and supportive antiseptic therapy with 0.2% Chlorhexidine rinses (Dentohexin, Streuli, Uznach, Switzerland) twice daily for 7 days. Patients were recalled after 10 days post-surgery for suture removal and after 5 months for CBCT scans and implant placement.

### 2.4. Implant Placement and Core Biopsy Removal

Five months after block augmentation, the alveolar ridge’s status was assessed regarding shape, volume, and soft tissue status during the intraoral examination, followed by implant placement (implant types, diameters, and sizes were chosen according to Table 1, Institut Straumann AG, Basel, Switzerland) comprising core biopsy removal. In brief, the alveolar crests were fully exposed by raising a mucoperiosteal flap, and bone block fixation screws were removed. Core biopsies were retrieved at the planned implant positions using a Ø3.5 mm trephine explantation bur (Straumann, Basel, Switzerland) under constant irrigation with sterile saline. The axial orientation of the biopsy cores was marked in situ at their crestal aspect relative to the axis of the alveolar ridge before removal as an orientation reference for histological processing. Next, the resulting osteotomies were extended to their final diameters and fine-prepared according to the manufacturer’s instructions. Healing abutments were mounted, and primary wound closure was accomplished (Supramid 4.0, B.Braun^®^, Melsungen, Germany) for transgingival healing. Implants were restored and loaded 2 months after placement with a screw-retained restoration obtained from conventional laboratory procedures.

### 2.5. Radiographic Measurements

The horizontal alveolar dimensions were evaluated using CBCT scans obtained before and 5 months after block augmentation. The evaluation was restricted to lateral augmented defects, excluding sinus-augmented areas, to improve defect type consistency. Horizontal dimensions in the buccopalatal direction were measured at planned implant positions along the implant axis or the mid-axial implant positions.

Marginal bone levels were measured as previously described [24]. In brief, mesial and distal crestal bone margins were individually assessed at the time of implant placement, time of loading, and 1 year after placement by visually determining the distance between the most coronal bone to implant contact relative to the implant platform from peri-apical radiographs (Romexis, Planmeca, Helsinki, Finnland). Radiographs were dimensionally calibrated using the implant length or platform diameter. Alveolar dimensional and marginal bone level values are reported as medians with the first and third quartiles.

### 2.6. Histology

The as-above-described obtained core biopsies (“carrots”) were immersed in a solution of 10% neutral buffered formalin for 1 week, decalcified in Osteosoft, dehydrated in ethanol/xylene, and embedded in paraffin wax. Sections of 5 μm were cut in mesiodistal direction along the longitudinal plane using a microtome and were stained using hematoxylin and eosin saffron (HES). Central sections were used for evaluation.

### 2.7. Case Series Setup

The clinical procedures, including block augmentation, core biopsy removal, implant placement, and radiographic alveolar dimensional evaluation, are illustrated for three representative cases.

#### 2.7.1. Case 1

Case 1 illustrates the reconstruction and restoration of a 54-year-old female referral patient displaying severe horizontal atrophy in position 14. Preoperative CBCT indicated a mean residual bone height of 13 mm between the alveolar crest and nasal cavity and an alveolar thickness in the vestibular–palatal direction of 6 mm. The consented treatment plan was based on a two-stage procedure comprising lateral alveolar augmentation with a bone block followed by implant placement.

**Figure 2:**
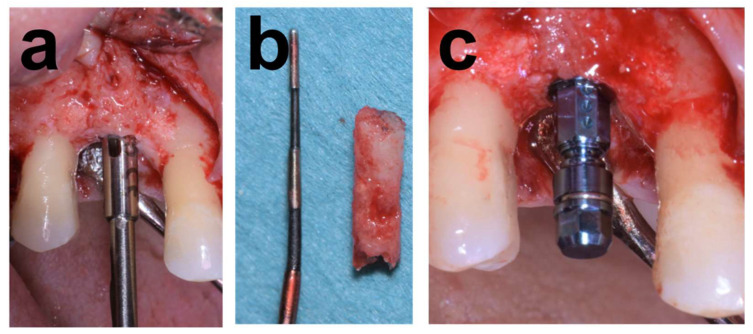
Representative illustration of core biopsy retrieval and implant placement in Case 1. (**a**) Preparation of the core biopsy from the augmented area using a trephine bur and (**b**) resulting core biopsy before histological processing. (**c**) Situation after placing a Ø4.1 × 12 mm implant in the osteotomy resulting from biopsy retrieval with transfer piece in place.

**Figure 3:**
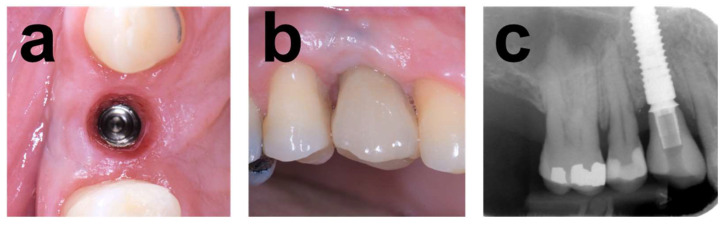
Follow-up documentation of Case 1. (**a**) Occlusal view 2 months after implant placement and after removing the healing abutment illustrates the adequate horizontal contours and dimensions of the regenerated alveolar crest and peri-implant soft-tissue conditions. (**b**,**c**) Lateral clinical view and peri-apical radiograph illustrating stable and adequate peri-implant soft tissue, implant integration, and stable peri-implant osseous conditions and marginal levels 3 years post-surgery.

**Figure 4:**
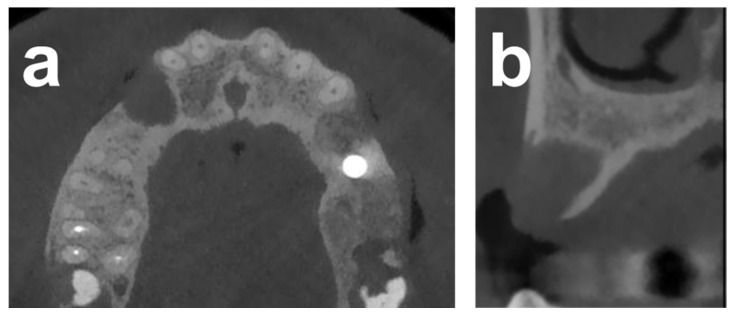
CBCT sections in 2D illustrate the horizontal alveolar dimensions in the defect areas (13 and 14) 3 months after tooth extraction. (**a**) Two-dimensional transversal and (**b**) frontal maxillary CBCT sections illustrating the severe alveolar defect.

The atrophy was associated with removing an implant placed in conjunction with alloplastic bone grafting affected by severe peri-implantitis. Figure 1 illustrates the baseline situation before and after debridement, curettage, and rinsing with NaCl and Clindamycin solution (Sandoz, Rotkreuz, Switzerland). The resulting horizontal alveolar defect extended 5 mm in mesiodistal and 10 mm in the peri-apical direction. Augmentation consisted of an allograft block adapted in size and shape to the defect anatomy and secured with an osteosynthesis screw. The lateral aspects around the block were augmented with allogeneous particles from block trimming (Figure 2), covered with a membrane and closed. The clinical situation in Figure 3 after 5 months of healing and reopening for second-stage surgery and the comparisons between baseline and 5-month follow-up CBCT scans in Figure 4 illustrate an adequate osseous integration and dimensional reconstruction of the alveolar dimensions. The resulting ridge contours allowed implant placement (Bone Level, Ø4.1 × 10 mm) within the bone housing with adequate primary stability after core biopsy removal of a histological sample (Figure 2). Figure 3a illustrates the resulting ridge contour and ideal soft tissue situation at the time of restoration, consisting of a screw-retained restoration obtained from conventional laboratory workflows. Marginal bone levels and soft-tissue conditions at the 3-year post-restoration re-entry were stable and adequate (Figure 3c).

#### 2.7.2. Case 2

Case 2 illustrates the alveolar reconstruction and implant restoration of a 53-year-old male referral patient with severe horizontal atrophy in positions 13 and 14 that was associated with the removal of an included canine, performed 3 months prior. Diagnostic CBCT indicated a residual vestibular–palatal alveolar thickness of 2 mm in the edentulous premolar area (Figure 4). The consented two-stage treatment plan comprised lateral alveolar bone block augmentation followed by implant placement in positions 13 and 14.

**Figure 5:**
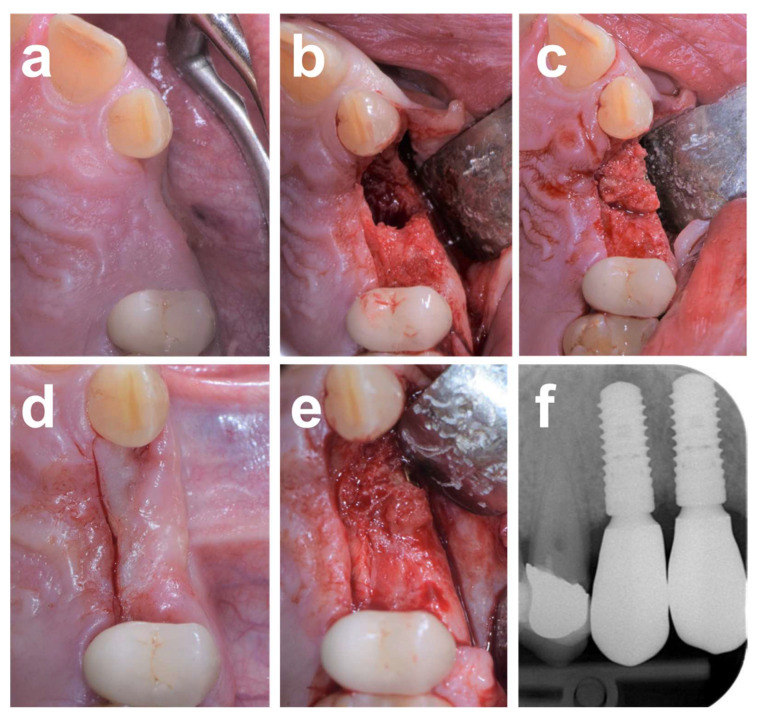
Treatment sequence of Case 2. (**a**) Occlusal clinical view prior to treatment. (**b**,**c**) Situation after midcrestal and laterally extended intrasulcular incision and buccal flap elevation exposing the alveolar defect and after lateral block augmentation and fixation using an osteosynthesis screw, respectively. (**d**,**e**) Occlusal view on the augmented area at 5 months after healing before and after midcrestal incision and full-thickness flap elevation, indicating adequate graft integration and reconstruction of the alveolar contour. (**f**) Peri-apical radiograph of the final restoration at the 3-year post-placement re-entry indicating adequate implant integration and stable peri-implant osseous conditions and marginal levels.

**Figure 6:**
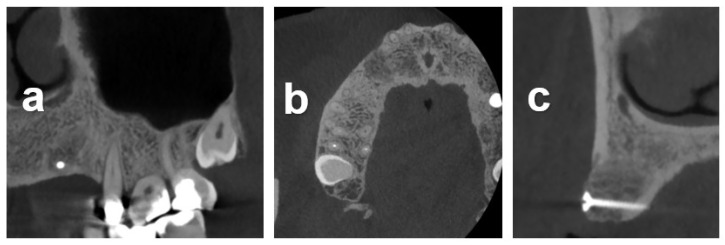
Sagittal (**a**), transversal (**b**), and frontal (**c**) 2D CBCT sections of the block-augmented area obtained 5 months after augmentation.

Figure 5 illustrates the baseline situation and treatment sequence, including defect exposure and allograft block augmentation secured with a temporary osteosynthesis screw. Allogeneous particles from block trimming were used to augment the lateral aspects around the block-augmented area, followed by membrane coverage and primary wound closure. The clinical situation after 5 months of healing and reopening for second-stage surgery, shown in Figure 5d,e, in conjunction with the follow-up CBCT at the corresponding follow-up time point, shown in Figure 6, indicated an adequate dimensional reconstruction of the alveolar dimensions. Implants (Bone Level, Ø4.1 × 10 mm) were placed in the ideal prosthetic position with adequate primary stability following removal of histological core biopsy samples.

Marginal bone levels and soft-tissue conditions at the 3-year post-restoration visit with screw-retained crowns were stable and adequate (Figure 5f).

#### 2.7.3. Case 3

**Figure 7:**
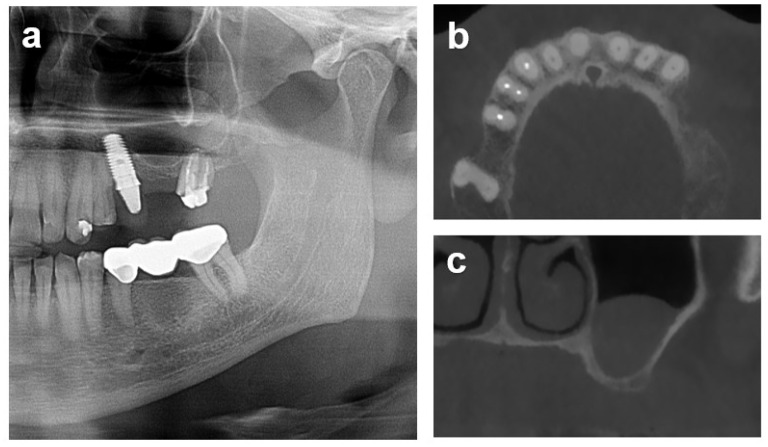
Case 3: Initial patient situation and baseline radiographic assessments of patient 2 three months after tooth and implant removal. (**a**) Pre-treatment panoramic radiographic assessment of the patient after removal of his left posterior restoration. Implant 24 was diagnosed with progressed peri-implantitis. Root remnants of abutment tooth 26 presented fractured, with a peri-apical pathology, and were indicated for extraction. (**b**) Two-dimensional transversal maxillary CBCT section illustrating the progressed horizontal atrophy in the distal aspect 3 months after implant and tooth removal. (**c**) Two-dimensional frontal plane in zone 26 illustrating the pronounced vertical alveolar atrophy of the distal maxilla after tooth removal omitting implant placement without prior augmentation. Membrane thickenings of the sinus floor, potentially associated with inflammatory processes at implant 24 and tooth 26, were confirmed to be non-pathological by a specialist and resolved spontaneously after extraction and explantation, respectively.

**Figure 8:**
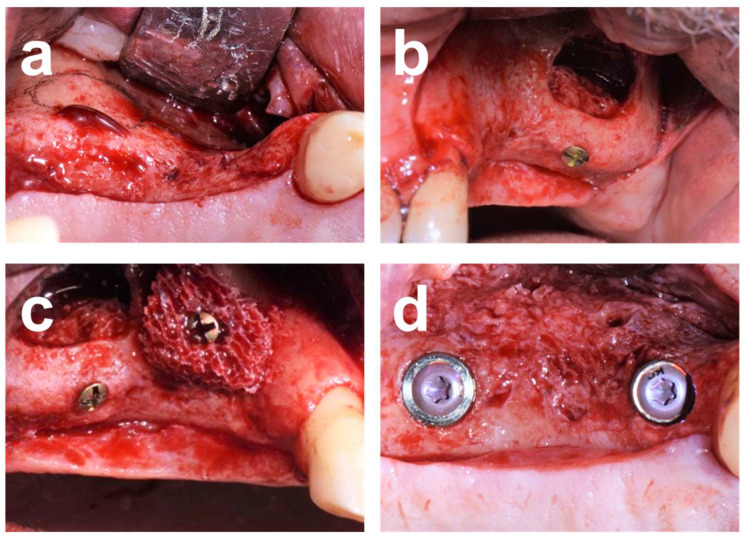
Treatment sequence illustration of Case 3. (**a**) Occlusal view after mucoperiosteal flap elevation exposing the pronounced horizontal vestibular alveolar atrophy in the premolar maxillary region. The position of the lateral window for the planned sinus floor elevation has been marked. (**b**,**c**) Combined lateral and sinus augmentation, including situations after anterolateral antrostomy, intra-sinus placement and fixation of a block graft, and lateral vestibular block augmentation and fixation in zone 26. (**d**) Occlusal view on the exposed augmented area 5 months after healing and after placement of 2 bone-level implants in positions 24 (Ø4.1 × 10 mm) and 26 (Ø4.8 × 10 mm) and installation of healing caps.

**Figure 9:**
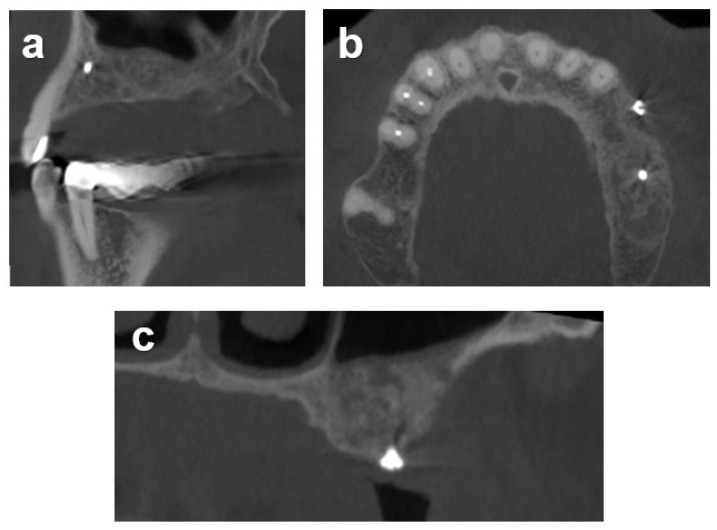
Situation 5 months after combined horizontal lateral onlay graft and vertical sinus augmentation illustrating the resulting horizontal and vertical alveolar dimensions as illustrated by corresponding sagittal (**a**), transversal (**b**), and frontal (**c**) 2D CBCT sections.

Case 3 illustrates a combined horizontal lateral onlay graft and vertical sinus augmentation in a 62-year-old male referral patient with a severely atrophied posterior maxilla following implant explantation and tooth-root removal. CBCT assessments revealed pronounced horizontal atrophy in positions 24 and 25 and a residual bone height of 2.5 mm in zone 26 (Figure 7). A staged combined sinus and onlay grafting with allograft blocks was used to reconstruct the posterior left maxillary ridge contour (Figure 8). CBCT analysis after 5 months of healing confirmed adequate horizontal and vertical osseous dimensions, allowing implant placement at the ideal prosthetic positions (Figure 9). Osteotomy preparation and implant placement after retrieving core biopsies for histological processing resulted in adequate primary implant stability with insertion torque values of 40 and 32 N/cm in the anterior and posterior implant positions, respectively (Figure 8d).

## 3. Results

**Figure 10:**
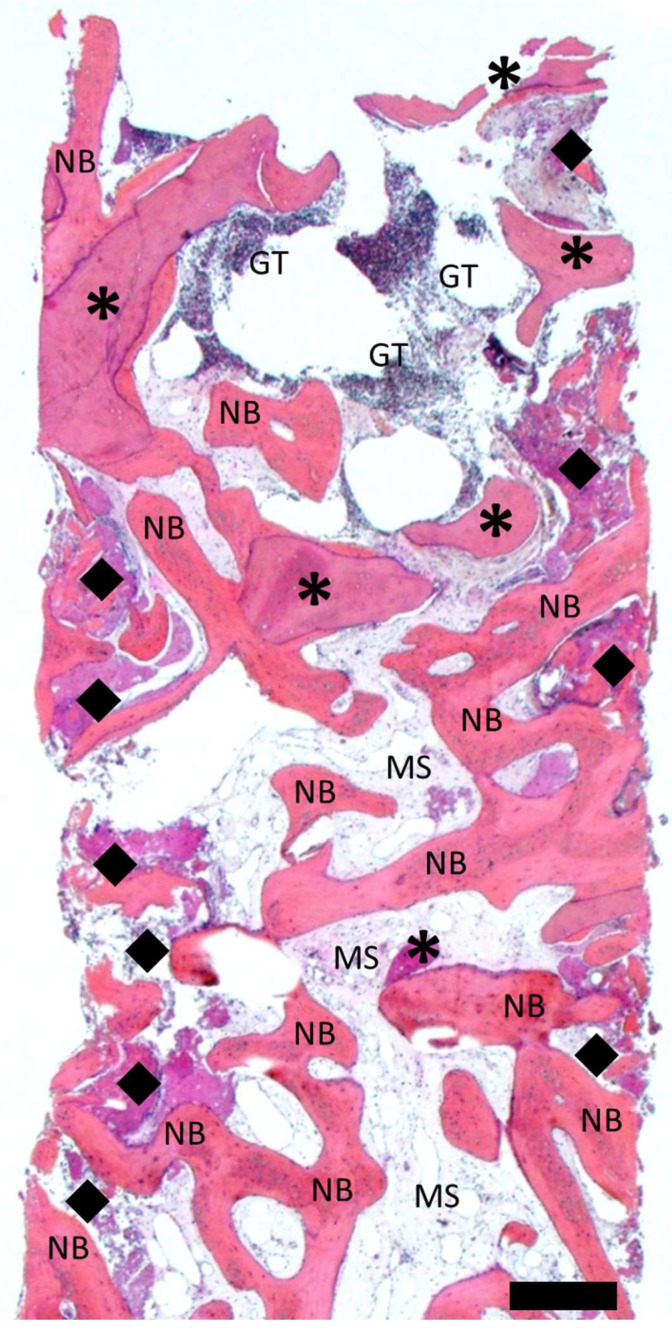
Overview of a representative core biopsy histological mesiodistal cross-section from Case 1. Bone graft remnants were stained with increased intensity (asterisks). Newly formed trabecular bone appeared slightly less intensely stained (N.B.) and separated by marrow spaces (M.S.). The remaining bone graft at the lateral aspects of the core biopsy was characterized by ongoing resorption and new bone formation (black diamonds) compared to the more intact remaining bone graft in the center of the biopsy. Zones of granulation tissue (G.T.) were apparent at the central coronal aspect of the biopsy. Scale bar: 500 µm.

**Figure 11:**
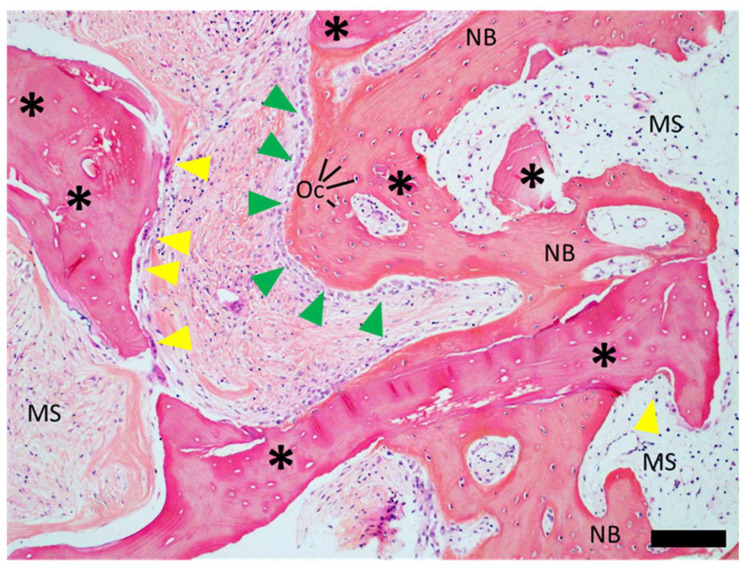
Representative high magnification histological cross-section of core biopsies (“carrot”) obtained from patient 2 illustrating the processes at the lateral aspects of the biopsies displaying bone ingrowth. The remaining bone graft was identified as comprising darker stained areas (asterisks), and newly formed trabecular lamellar bone as brighter pink structures (N.B.) intercalated with numerous marrow spaces (M.S.). Newly formed bone was characterized by osteocytes (Oc) while the remaining graft displayed empty osteocyte lacunae. Osteoclasts were found at the surface of the remaining bone graft (yellow arrows), while osteoblast seams indicated active bone formation at the newly formed trabecular bone surface (green arrows). Marrow spaces occasionally showed the presence of fibrous tissue. Scale bar: 200 µm.

The procedural aspects and clinical observations are illustrated in Figure 1, Figure 2, Figure 3, Figure 4, Figure 5, Figure 6, Figure 7, Figure 8 and Figure 9. Figure 10 and Figure 11 illustrate the histological findings, representatively, of the collated histological micrographs provided in Supplementary Figure S1. The individual and average crestal dimensions before and after augmentation and the temporal evolution of marginal bone levels for all five patients are reported in Table 1 and Table 2. Supplementary Figure S2 provides an overview of the different treated defect types of the individual patients.

### 3.1. Clinical Results

Healing after block augmentation and implant placement was uneventful in all cases. For all treated patients, lateral or combined lateral and sinus augmentation yielded adequate alveolar dimensions to allow implant placement in the ideal prosthetic positions with sufficient primary stability between 30 and 45 N/cm. In all cases, augmented bone blocks appeared well integrated into the alveolar process and clinically stable and consistent. None of the grafts displayed signs of infection, necrosis, or wound dehiscences. All implants could be successfully restored and loaded and were followed up by clinical and radiographic examination for at least 1 year. All implants presented healthy and without any pathological signs, based on the absence of signs of inflammation, bleeding, redness, swelling, mobility, or patient-reported symptoms comprising pain, discomfort, or changes in sensation at the 1-year follow-up. Furthermore, marginal bone levels around all implants were optimal and stable at this follow-up.

### 3.2. Radiometric Assessment

In all patients, a pronounced and adequate CBCT-derived horizontal bone gain could be identified, increasing the median horizontal alveolar dimensions from a median of 3.0 (2.3 to 4.5) mm prior to treatment to 10.8 (10.0 to 11.9) mm at the time of implant placement. The median increase was 7.0 (5.5 to 7.8) mm (Table 2).

The radiometric comparison of the post-loading and 1-year follow-up peri-apical radiographs revealed constant marginal bone levels at the implant platform for all lateral-block-augmented sites. Variations in individual results between both time points remained small and unpronounced (Table 2). The only site with a detectable change in the marginal bone level was site 26 in patient 4, which was sinus- but not lateral-block-augmented and attributed to changes in native crestal bone. The resulting mesiodistal median marginal bone-level change 1 year after implant placement was 0.0 (−0.1 to 0.0) mm.

### 3.3. Histological Characterization

Figure 10 and Figure 11 show representative histological cross-sections of Cases 1 and 4, illustrating the overall tissue graft integration and detailed processes at the graft tissue interface at the lateral inferior aspect of the graft. Histological observations in all cases were consistent with regard to the overall pattern of tissue integration and graft host reactions (Supplementary Figure S2).

As evidenced by the histological overview in Figure 10, the newly formed bone and remaining allograft appeared distinguishable by differences in the staining intensity. Newly formed bone intercalated with marrow spaces was present throughout the full extent of the biopsy. The magnitude and integrity of the remaining bone graft varied between the lateral and central aspects of the biopsies. The remaining graft appeared relatively preserved, intact, and well integrated into the newly formed bone in the center and apical aspects of the biopsy (“carrot”). In the lateral aspects, however, the remaining graft appeared progressively resorbed and replaced with new bone. The center–coronal aspect of the biopsy displayed a protruding zone of granulation tissue potentially associated with ongoing inflammatory processes. No signs of inflammation or granulation tissue were found in the central and apical aspects of the biopsies.

Histomicrographs at higher magnification revealed a distinct mature lamellar structure of newly formed bone and the presence of osteocytes (Figure 11). Osteoid seams on the surface of newly formed bone indicated active ongoing bone formation. The remaining bone graft, which could be well distinguished from newly formed bone by the consistent presence of empty osteocyte lacunae, was well integrated into the newly formed bone at the lateral aspects of the biopsy. Central aspects of the biopsy showed signs of active ongoing resorption as indicated by graft-surface-associated osteoclasts. Marrow spaces were, in places, fibrotic.

## 4. Discussion

The present study clinically and histologically investigated the performance of commercially available allogeneic cancellous bone blocks (Maxgraft^®^) to horizontally and vertically augment local alveolar defects for implant placement. Considered treatments comprised a range of different alveolar defect morphologies at different anatomic sites in healthy patients without known conditions influencing treatment outcomes. The dimensions of the alveolar crest before and 5 months after block augmentation were radiographically evaluated. Alveolar bone gain was contextualized with histological graft integration, resorption, and new bone formation.

Few case reports and clinical studies have so far been published that employed the study graft material in particulate form or as block grafts, with the latter being usually more prone to the presence of tissue remnants [6,18,20,21,22,23,27]. To the best of our knowledge, the present report is the first to describe the clinical and biological performance of the study material in block graft form for lateral or sinus augmentation as part of two-staged implant treatment procedures in a series of patients.

The median horizontal alveolar bone gain after 5 months of healing was 7.0 (5.5 to 7.8) mm. These values are comparable with literature reports employing comparable block graft materials. Specifically, Nissan et al. reported between 5 and 5.6 mm of horizontal bone gain 6 months after grafting with cancellous allografts and a nonsignificant 0.5 mm post-augmentation surface resorption of the grafted ridge [28,29,30]. These results were high compared to the horizontal bone gain of 4 mm using a cortico-cancellous FDBA, reported by Ahmadi et al., or of 3.5 mm using DFDBA cortico-cancellous bone, reported by Toscano et al. [5,31]. Comparisons with these values also need to consider that cortico-cancellous blocks were reported to display smaller resorption rates than the cancellous bone graft types used herein [32]. It also needs to be acknowledged that the results might be affected by the vertical position of measurement relative to the bone crest, which, in our study, was chosen mid-axially to the planned implant as a clinically relevant position.

It is also interesting to compare the herein reported results with values reported by Solakoglu et al. as they allow a comparison between granulate and block grafts of the study-type material under the consideration of potential limitations associated with differences in the study setups and clinical procedures and the differences in the number of treated patients [6]. Specifically, Solakoglu et al. reported an average increase of 2.29 mm in alveolar ridge width. This lower value compared to the results found in our study may further support the described superior efficacy of bone block grafts over granulate materials for horizontal augmentative procedures [2,6].

Although a vertical bone gain was also reported with cancellous bone graft blocks, vertical augmentations were only performed in two patients as part of sinus grafting in this report, rendering the sample cohort small and inhomogeneous for a meaningful comparison with the literature values [28]. All analyzed defect types were classified as three-wall defects, with a vertical component that strictly cannot be considered as indicating vertical defects. Consequently, analysis and contextualization were mainly restricted to the horizontal alveolar dimensions.

Marginal bone levels were also followed up over a time period of 1 year post-surgery, indicating overall stable marginal bone levels and an overall median loss of 0.0 (−0.1 to 0). Furthermore marginal bone loss was mainly attributed to a loss of native but not block-augmented bone. The observed stable marginal bone levels are consistent with the results reported by Solakoglu et al.. Specifically, the authors reported stable marginal bone levels over 3 years after implant loading following a two-stage augmentation implant procedure using the same type of material investigated in this study in particulate form. To our knowledge, this was the first study that reported marginal bone levels after block augmentation with the herein-investigated material. It is also interesting to note that marginal bone levels in block-augmented bone analyzed herein were stable despite a potential for block surface resorption, which was reported by some authors [5]. The latter was primarily described for the first 6 months after block augmentation and its magnitude was reported to vary quite strongly [5,30]. Further studies may be necessary to elucidate this process’s magnitude and temporal evolution for the studied material and its potential effect on marginal bone levels in more detail.

From a mechanistic point of view, the histological analysis revealed that new bone ingrowth and the graft integration of the bone block were found to take place from the lateral and apical aspects. Central and coronal aspects of the allogenous graft appeared intact and were characterized by soft tissue infiltration. This result was in agreement with previous histological reports describing bone blocks as a space-holding scaffold for the ingrowth of bone via osteoconduction lacking full remodeling [5,19]. Despite this apparent lack of internal and coronal bone formation, all bone blocks integrated well into the alveolar crest and provided an adequate and mechanically stable framework to provide appropriate levels of primary implant stability after 5 months of healing.

Crestal mucosal remnants on the basal edge of the graft were characterized by signs of moderate immunologic cell infiltration and granulation tissue formation, which was also reported by other authors [21]. It is also interesting to note that previous histological studies on cancellous lateral bone grafting procedures revealed that patient age strongly influenced new bone formation, with patients below 40 showing almost twice the amount of newly formed bone compared to patients above 40 years [33]. The patient cohort of the present investigations had an average age of 60 (53 to 68) years and thus exclusively comprised patients belonging to the age group of patients with potentially delayed bone formation. Despite these potentially less favorable patient demographic characteristics, the current investigation did not indicate any negative impact of the moderate patient age on the studied clinical outcomes. Studies addressing the histological bone formation with larger patient cohorts may be necessary to confirm or disprove a potential impact of age on the here-described study setup and materials.

Another histological finding was related to the presence of multinucleated giant cells (MNGCs) at the graft material’s surface. This finding was in agreement with a recent clinical, histological investigation by Lorenz et al., who studied the tissue reaction and bone formation of a different allogeneic spongious bone block (Tutobone^®^, Tutogen Medical, Neunkirchen, Germany) [19]. No immunohistochemical analysis was applied herein to characterize the MNGCs regarding, e.g., the expression of tartrate-resistant alkaline phosphatase (TRAP). Nevertheless, the results reported herein seem to be in good agreement with the observation of Lorenz et al. with regard to the observation that the presence of MNGCs did not appear to influence bone formation [34].

Interestingly Solakoglu et al. could not identify foreign-body-associated MNGCs when studying the granular variant of the used study graft material. The authors attributed this finding to the higher efficiency of decellularization of granular materials compared to block allografts. The here-reported presence of MNGCs compared to the results reported by Solakogly et al. might indirectly confirm this assumption for this specific type of material [23].

Another aspect of the study was related to the use of cancellous blocks for both kinds of procedures, i.e., lateral augmentations and sinus augmentation. Chaushu et al. recently reported a success rate of 95% for procedures using cancellous bone blocks for sinus augmentation in conjunction with simultaneously placed implants. While this procedure may be regarded as more demanding than the herein-described one, it demonstrates both the suitability of cancellous bone graft materials for this indication and their ability to enhance implant stability even prior to integration [35]. More recently, the same authors reported that the radiographic bone gain and histological bone formation as part of implant placement in simultaneously augmented sinuses were comparable between allogeneic cancellous bone blocks and allograft particulate materials [36]. Despite the limitations related to the limited number of cases, documenting this indication herein, to the best of our knowledge, the current report is the first to describe the use of the investigated material in the form of cancellous bone FDBAs as part of sinus-block-augmentative procedures.

In addition to the mentioned aspects, the authors want to emphasize that the type and biological properties of the block graft material need to be evaluated in conjunction with several other determinants potentially influencing clinical outcomes. These factors may comprise the stabilization and intimate contact of the graft block to the osseous walls of the recipient site; defect morphology; site location, i.e., maxilla vs mandible; the site preparation comprising decortication to provide access by osteoprogenitor cells; and the use of barrier membranes [2,3,4,5,7,37,38].

From a broader perspective, the results herein support the use of block allografts for the staged lateral alveolar augmentation as valuable potential alternatives to conventional GBR procedures combining granulate materials and membranes. Recent systematic reviews indicate that both modalities result in high implant survival rates and long-term crestal bone stability [7,39,40,41]. Outcomes and comparative assessments of both techniques may strongly depend on the exact indication, defect anatomy, timing of the procedure, and applied techniques [7,39,41]. Regarding the latter aspect, block grafting was considered in the reported cases due to the advanced defect dimensions and the potential advantage of block grafts of remaining more stable and robust against graft displacement upon wound closure and healing. There is a lack of evidence supporting the use of barrier membranes with bone block grafting [41,42].

Despite the overall consistency with the applicable literature, the herein-reported observations and interpretations are tempered by the limited sample size, the pilot character of the chosen study setup, and the variations in treated defect-type morphology. Furthermore, it must be acknowledged that this report consistently documented the reconstruction of three-wall-type defects in the maxillary premolar region. Thus, the reported observations may strictly apply exclusively to this specific indication. To further substantiate and investigate the reported effects, follow-up studies with larger patient numbers and different bone graft materials as controls might be considered.

Future aspects may also include onlay bone graft customization, which may further help to reduce surgery time and patient morbidity while increasing the contact between the graft and the alveolar bone. Venet et al. described this approach as part of a case series [43]. However, the application of more advanced procedures like combined lateral and sinus augmentations has not been demonstrated so far. Bone graft customization could, therefore, be a modality to further increase the potential of allografts in achieving similar clinical outcomes to autografts.

## 5. Conclusions

Cancellous freeze-dried bone allografts allowed for pronounced and adequate alveolar horizontal and vertical bone gain as part of two-staged implant therapy for patients with local alveolar atrophies. The resulting alveolar dimensions and contours allowed implant placement in the ideal prosthetic positions with optimal primary stability despite ongoing biologic graft integration. Although block grafts may contain potentially immunogenic cell and tissue remnants, histologically identified foreign-body reactions of the study material could not be correlated to any clinical performance losses or adverse reactions. Within the study’s limitations, these results support using the studied cancellous freeze-dried bone block allograft as a suitable material for the lateral augmentation of local alveolar defects.

## Figures and Tables

**Table 1:** Patient demographic and treatment-related information.

			Patient		
	1	2	3	4	5
Gender	F	M	M	M	F
Age	54	53	68	63	62
Treatment positions	14	13, 14	27	24, 26	14, 16
Implant Type	BL RC	BL RC	TL WN	BL RC	TL WN
Implant Dimensions	Ø4.1 × 12 mm	Ø4.1 × 10 mm Ø4.1 × 10 mm	Ø4.8 × 10 mm	Ø4.1 × 10 mm Ø4.8 × 10 mm	Ø4.1 × 10 mm Ø4.8 × 10 mm
Insertion Torque [N/cm]	45	35, 40	30	40, 32	35, 30

Abbreviations: F: Female, M: Male, BL: Bone Level Implant, TL: Tissue Level Implant, RC: Regular Colar, WN: Wide Neck, H: Horizontal defect, and V: Vertical defect.

**Table 2:** Horizontal and vertical alveolar dimensions at the treatment sites, i.e., sites of implant placement, before (baseline) and 4–5 months after block augmentation and resulting horizontal and vertical bone gains derived from Cone Beam Computer Tomographic (CBCT) sections. Marginal bone levels after treatment and at follow-up, i.e., at and after 1 year of implant placement, are shown. Dimensions are reported in mm. Values are reported as medians and ranges denoting the 1st and 3rd quartiles and as absolute ranges reporting the minimum and maximum values. Abbreviations—IQR: Interquartile range.

			Patient				
	1	2 ^†,§^	3	4 ^†^	5 ^†,††^	Median (1st to 3rd IQR	Range
**Horizontal Dimensions (CBCT) [mm]**							
Baseline	3.0	2.0	2.0	5.0, 8.4	3.0	3.0 (2.3 to 4.5)	2.0–8.4
5-month follow-up	8.0	10	11.5	12.0, 12.5	10	10.8 (10 to 11.9)	8.0–12.5
Horizontal Gain ^‡^	5.0(×9.0) ^‡^	8.0(×9.0)	9.5(×8.0)	7.0 (×11.0), 4.0 (×11.0)	7.0(×5.2)	7.0 (5.5 to 7.8)	4.0–9.5
**Marginal Bone Level (2D) [mm]**							
Post-OP mesial	0.0	0.0, 0.3	0.2	0.0, 0.0	0.2	0.0 (0.0 to 0.2)	0.0–0.3
1-year mesial	−0.1	0.0, 0.3	0.0	0.0, −1.2	0.0	0.0 (−0.1 to 0.0)	−1.2–0.3
Post-OP distal	0.0	0.3, 0.0	0.0	0.0, 0.0	0.0	0.0 (0.0 to 0.0)	0.0–0.3
1-year distal	0.0	0.3, −0.0	0.0	0.0, −1.2	0.0	0.0 (0.0 to 0.0)	−1.2–0.0

‡ Values in parentheses report the vertical extent of the defect over which the horizontal, i.e., lateral, gain was observed, reporting the resulting cross-sectional areas of the augmented zones, e.g., 5.0 mm in the horizontal direction over a distance of 9.0 mm in the vertical direction for patient 1. † Values for patients 2, 4, and 5 refer to the indicated individual treatment positions. § Alveolar dimensions for patient 2 are reported only for implant 13, which was center-positioned in the augmented area. †† Alveolar dimensions and marginal bone levels for patient 5 were reported for position 14, which was laterally block-augmented, as opposed to the sinus-augmented position 16.

## Data Availability

Data are contained within the article.

## Supplementary Materials

The following supporting information can be downloaded at: https://www.mdpi.com/article/10.3390/dj12020042/s1, Figure S1: Collated transversal CBCT sections of Patients 1 to 5 illustrating comparable and consistent baseline defect morphology and defect location between individual patients. The upper and middle rows illustrate the baseline defect morphology as 3 wall horizontal defects in all patients. The lower line documents the 5 months post-block augmentation CBCT follow-up. Figure S2: Collated histological cross section overviews (upper row) and magnified regions (lower rows) of core biopsies of patients 2 to 5. Scale bar in the overview and magnifified images designate 500 and 200 µm, respectively. The corresponding images of patient 1 are shown in the main part of the manuscript.
