# Supplementary material for: Clinical and Biological Validation of an Allogeneous Cancellous Bone Block for Alveolar Maxillary Ridge Reconstruction: A Case Series
Source: Dent J (Basel). 2024 Feb 19;12(2):42. doi: 10.3390/dj12020042 (PMC10888231; doi:10.3390/dj12020042)
Supplement: Supplementary file 1 [file dentistry-12-00042-s001.zip › dentistry-2871414-supplementary.pdf]

Supplementary Information

Figure S1

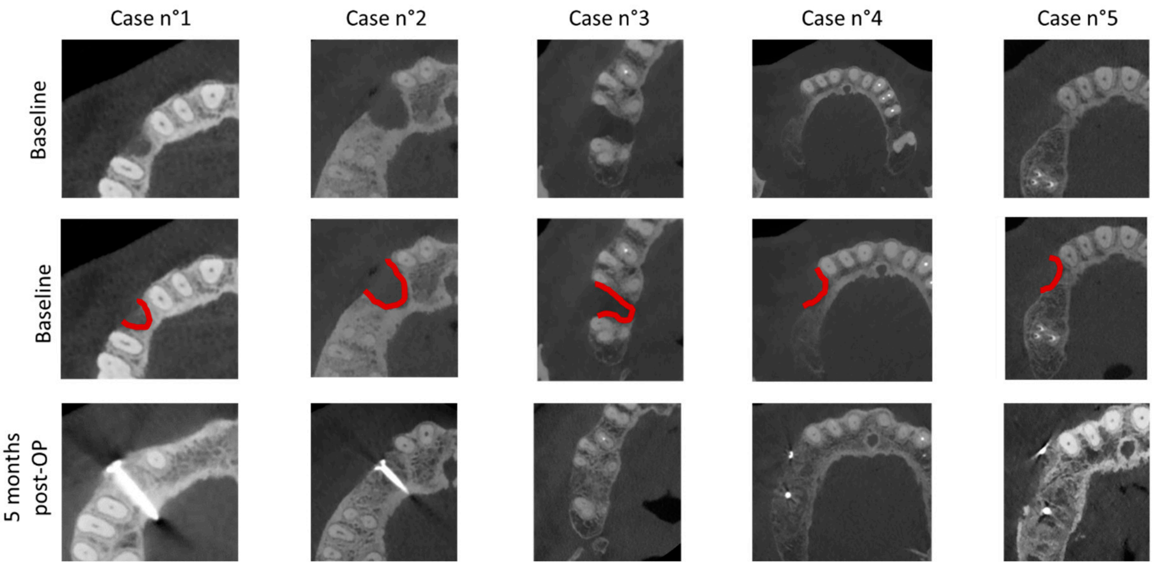

Figure S1: Collated transversal CBCT sections of Patients 1 to 5 illustrating comparable and consistent baseline defect morphology and defect location between individual patients. The upper and middle rows illustrate the baseline defect morphology as 3 wall horizontal defects in all patients. The lower line documents the 5 months post-block augmentation CBCT follow-up.

Figure S2

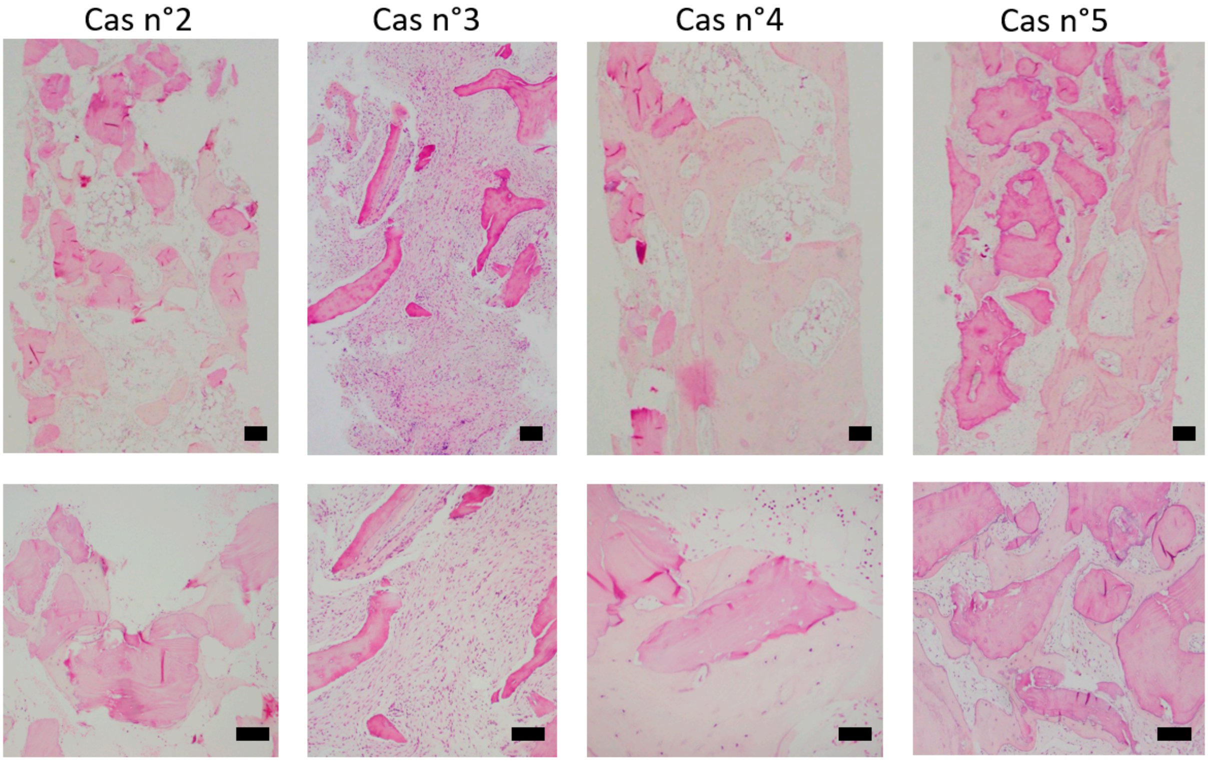

Figure S2: Collated histological cross section overviews (upper row) and magnified regions (lower rows) of core biopsies of patients 2 to 5. Scale bar in the overview and magnified images designate 500 and 200 µm, respectively. The corresponding images of patient 1 are shown in the main part of the manuscript.
